# Rabies and Hydrophobia1Read at the Thirty-seventh Annual Meeting of the American Veterinary Medical Association, Detroit, September, 1900.

**Published:** 1900-10

**Authors:** D. E. Salmon

**Affiliations:** Chief of Bureau of Animal Industry


(To be continued.)

---

### RABIES AND HYDROPHOBIA.[1]

By D. E. Salmon, D.V.M.,
CHIEF OF BUREAU OF ANIMAL INDUSTRY.

THERE has, perhaps, never been a time when it was more important than it is to-day for the veterinary profession to have a clear appreciation of what it knows in regard to the subject of rabies in animals and in man. In the pre-experimental period of the profession's existence there was ample excuse for difference of opinion, even, concerning the most apparent and most essential phenomena of this disease. It was the time when dependence was placed upon clinical observation of accidental cases, where the conditions were not controlled, and where the conclusions founded upon imperfect evidence were often unsound and liable to vary with the idiosyncrasies of the observer; but that period is past. To-day we have a science resting upon an experimental basis. Facts and conclusions have been established just as rigorously, just as solidly, as in other departments of science; and while we still have much to learn, as they have in every other branch of science, the fact that there are other fields to conquer does not detract in the least from the value or certainty of the results which have already been achieved.

As members of a learned profession it is our duty to know what has been accomplished by the scientific investigation of rabies; and particularly is this duty incumbent upon those who attempt to teach other members of the profession or the laity as to the facts in the case or the attitude of the profession. We have reached a point where the intelligence, the scientific knowledge, the standing of the veterinary profession in this country—I might say of the medical profession as well—are liable to be unjustly questioned because of a few mistaken and misguided individuals who persist in reiterating beliefs which never were held by a majority of the profession and which were discredited and disproved years ago.

[1] Read at the Thirty-seventh Annual Meeting of the American Veterinary Medical Association, Detroit, September, 1900.

The mistaken views to which reference has just been made may briefly be enumerated as follows:

1. There is no specific disease to which the term rabies can be applied. This alleged disease has not been clearly defined, and no characteristic features have been enumerated by which it can be identified. The supposed cases of rabies are ordinary affections, such as indigestion, epileptic fits, cerebritis, paralysis, etc., improperly diagnosed.

2. There may be such a disease as rabies, but it is extremely rare. Nearly all the cases alleged to be rabies are ordinary diseases improperly diagnosed. "Not one dog in a million supposed to be rabid is really affected with that disease," says one writer, and this opinion is echoed by the editor of a prominent medical journal.

3. The method of diagnosis by the inoculation of rabbits and guinea-pigs is uncertain and misleading. The same results may be obtained by inoculating with inert substances.

4. Admitting that rabies of the dog may be encountered in extremely rare instances, the disease is, nevertheless, not communicable to man. The alleged rabies of man is a nervous affection developed as the result of worry and fear after being bitten; it is really lyssophobia.

5. The destruction of superfluous dogs and the muzzling of dogs for the control of rabies are cruel and unnecessary measures.

It appears to the writer simply astounding that educated men, much less physicians and veterinarians, will put forward such propositions as these in regard to a disease which has been known and described in every age since the faintest dawn of history, and which for a century has been the subject of experimental investigation by the ablest men who have occupied their minds with pathological questions. Nevertheless, it is a fact that the sanitarian of to-day who tries to control rabies is obliged to meet the same kind of arguments which were used to embarrass his predecessors of one, two, and three centuries ago. These arguments are most industriously circulated by the so-called humane societies, which oppose all conclusions based upon experiments with animals, and which foolishly imagine that they are doing a great work for the dogs and cats by casting discredit upon science, even though by so doing they perpetuate this horrible disease of which dogs and cats are the principal victims. But, unfortunately, the humane societies and the lay press are not alone in this effort to discredit science. It was said a few years ago that there had been so much of this sort of literature circulated among physicians that many hesitated to return a death as from rabies when the history and symptoms of the case warranted that diagnosis. This false doctrine has been propagated to such an extent that in one of the great cities of the country, recognized as a centre of medical learning, there have been two coroners in succession skeptical as to the existence of hydrophobia in mankind, and who have refused to record deaths as occurring from this disease even when the cases were reported as such by the attending physician. In one such case I understand the diagnosis was proved by the inoculation of rabbits, and that the disease was transmitted to three series of these animals. In another and more recent case it is reported that an eight-year-old child was bitten in the ear by a tramp dog which strayed into the kitchen of her home. The wound soon healed, and nothing was thought of it until about six weeks later, when the child complained of pain in the head and neck, which was soon followed by difficulty of drinking, spasm of the phayrngeal muscles upon the slightest draught of air or touch of water upon the body or suggestion of drinking, great nervousness, with paroxysms of excitement and fear. Death occurred in about two days. Nevertheless, the coroner was positive, according to press reports, that the disease was not hydrophobia. How did he know?

In recent years we find a very few veterinarians in this country who practically agree with the humane societies, and either deny the existence of rabies or contend that if the disease exists it is so rare as not to be deserving of attention. The existence of such opinions in our profession emphasizes the importance of investigations to collect the facts and of discussions to fix these facts in the minds of our confrères.

It is not many years since the effort was made to eradicate contagious pleuro-pneumonia from this country, and then we were met by the same class of reckless assertions, to the effect that there was no pleuro-pneumonia in the country; that pleuro-pneumonia of cattle was not contagious, and that we might kill all the cattle in the country, and when we stocked up again we would have the same disease, since it was due to the conditions of environment and not to contagion. How completely all of these assertions have been disproved is known to every one of you. Again, it is not many years since an estimable army surgeon was presenting papers before our medical associations to prove that there was no such disease as Texas fever. To-day Texas fever is so well known that no one would undertake to question its existence and specific nature.

Of late years it has been the existence and specific nature of rabies that has been doubted and questioned and ridiculed.

*The Reality of Rabies.* What do we know about the existence of rabies, and how do we know it? Let us briefly inquire.

From the time of Aristotle (322 B. C.) until the present day we have clear accounts of this disease existing through every age, and provoking fear and horror in many countries. It was always caused by the bite of an animal, which animal was generally alleged to be rabid. It was almost invariably described as fatal in men and animals. The symptoms from the earliest times have been given as nervousness, restlessness, fear, irritability, paroxysms of fury, spasmodic contractions of certain muscles, paralysis, and death. Aristotle admitted that the disease was fatal to dogs and every other creature which they bite, except mankind. This early mistake in regard to the immunity of man has been carefully handed down across the centuries, and is still repeated on every hand by those who oppose measures for the prevention of the disease. It may be freely admitted, therefore, that there have probably been many in all ages who have doubted the existence of the disease both in mankind and in animals; that numerous articles and books have been written to prove that the disease called rabies was not contagious, and that the supposed rabies of man was lyssophobia—a nervous affection brought on by fear and excitement.

The medical profession as a whole has, however, always recognized the existence of such a disease as rabies in man. The veterinary profession has from its foundation recognized the existence and contagiousness of the disease; its schools from the earliest to the latest have consistently taught this doctrine, and its text-books are all but unanimous on the subject. Would it not be wonderful, amazing, incredible, if at this late day it could be proved that the thousands and hundreds of thousands of observations recorded from the birth of history to the present day were misconceptions; that the authors were deceived, and the disease a myth? Where can you find a parallel to such a complete overthrow of an ancient and almost universally accepted conclusion concerning a phenomenon so accessible to observation and investigation?

The doubts raised from time to time concerning rabies and its characteristics were, however, early met by scientific experiments. Zinke,[1] in 1804, announced that he had inoculated a dog, a rabbit, and a cock with saliva from a rabid dog, taking the saliva with a

[1] Zinke, Gottfried. Neue Ansichten der Hundswuth, etc., Jena, 1804, S. 180. Quoted by A. Högyes, Lyssa, Wien, 1897, p. 32.

brush from the animal soon after its death and spreading it over superficial wounds of the inoculated animals. The dog was inoculated in an anterior limb, and showed prodromic symptoms on the eighth day and was rabid on the ninth day. The rabbit was rabid on the eleventh day and the cock on the fourteenth day. This experiment, made so early in the century, proved that the disease of the dog called rabies was communicable by inoculation to the dog, the rabbit, and the fowl. It proved it to be a specific disease, and that the virus existed in the saliva. In this it confirmed the results of clinical observations extending over many years.

Count Salm-Reifersheid,[1] in 1813, recorded experiments in which several dogs were inoculated partly with fluid and partly with dried saliva from a rabid dog. These became affected with rabies after eight to ten days.

Berndt,[2] in 1822, inoculated four wethers with saliva from the mouth of an ox which had died of rabies. The inoculation was made by smearing the saliva over a wound made by an incision through the skin of the limbs. All of these sheep contracted rabies, the incubation period being twenty-two, twenty-five, twenty-six, and thirty-one days.

Hertwig[3] inoculated fifteen dogs during the years 1823, 1824, and 1825. The material used was saliva from rabid animals, and this was placed in lancet punctures made in the skin of the frontal region, except with two animals, in which the inoculation was made at the back of the neck. Six of these dogs contracted rabies, the periods of incubation being nineteen, twenty-seven, thirty-two, thirty-four, thirty-nine, and forty days.

On June 14, 1841, Professor Rey, of the veterinary school of Lyons, France, allowed a dog, brought to the hospital, affected with rabies, to bite a ram on the nose and right foot. The wounds cicatrized in five days. In thirteen days (June 27th) the animal showed the first symptoms of rabies. The following day it became furious when approached, violently attacked objects presented to it, threw itself against the wall and the bars of its pen, or rushed upon persons who came near it. It seized its chain with its teeth, attacked another sheep placed near it, butted it, then jumped upon it as if for copulation. The third day of its illness it was very weak and unable to rise. The fourth day it died.

1 Allgem. Anzeiger, 1813, No. 1. Quoted by Högyes, loc. cit., p. 32.

2 Journal der practischen Heilkunde, C. W. Hufeland, November, 1824, pp. 59-61.

3 Beiträge zur nähern Kentniss der Wuthkrankheit odor Tollheit der Hunde, vom Oberthierarzt Dr. Hertwig. Journal der practischen Heilkunde von C. W. Hufeland, 1828, pp. 112-129.

Two days before the death of the ram mentioned (June 28th) two other rams were inoculated from it. One had the lips rubbed several times against those of the affected animal, and the other was inoculated in the nose and upper lip by punctures of a lancet charged with saliva. The former did not contract the disease. The latter was first affected August 6th, or thirty-eight days after inoculation. The symptoms were similar to those of the first ram, and resulted in death.

December 21, 1841, a sheep which approached the kennel of a rabid dog at the Lyons school was seized by this animal and bitten through the nose. January 4, 1842, or fifteen days after inoculation, it showed the first sign of rabies. It died the third day. The second day of the disease saliva from this sheep was inoculated upon another sheep by lancet punctures at the nose and internal aspect of the upper lip. This sheep became affected January 27th, or the twenty-second day, and died the night of January 30th.

January 30, 1842, the last day of the life of the sheep just mentioned, its saliva was inoculated by lancet punctures in the nose and lip upon two other sheep. The first contracted rabies in twenty-three days, dying two days later; the second became affected in thirty-six days, and died in three days.

March 8th a sheep was inoculated from the last-mentioned animal and in the same manner as already described. April 15th it presented the symptoms of rabies after thirty-eight days' incubation. It was at first violent and later paralyzed in its posterior extremities; it died on the ninth day.

April 20th a sheep was inoculated with the saliva of the one last mentioned. June 1st, or forty-two days after inoculation, it presented symptoms of rabies of greater intensity than the one from which it was inoculated. It showed marked excitement, striking the soil violently with its forefeet when approached; when set free it ran after people, trying to strike them with its head, and attacked dogs particularly. It lived six days, complete paralysis of the posterior limbs occurring before death.[1]

A lamb was inoculated from the last of the series, which did not contract rabies, so that out of eight inoculations of sheep by biting or lancet puncture the disease was transmitted seven times.

These successful experiments made during the first half of the century by competent men are absolutely conclusive as to the existence of a disease of the dog communicable to dogs and to other

[1] Rey. Expériences sur la Rage. Journal de Médecine de Lyon, December, 1842, p. 461.

animals by biting and by inoculation with the saliva. If this disease is not rabies, what is it? And if you give it some other name do not the facts stand the same under one name as under another?

It is not correct to say that the disease alleged to be rabies has not been defined with sufficient clearness for its identification. Let us see: A disease affecting principally the nervous system, shown by restlessness, irritability, fury, convulsions, paralysis, death; caused by the bite of an animal similarly affected; communicable by inoculation with the saliva; having a long period of incubation (three to six weeks); comparatively short course of disease (two to ten days); invariably fatal. Is not that picture clear enough for identification? With what other disease can it possibly be confused? Let those who make these allegations in general terms descend for a moment to details and answer these questions.

Further, it is hardly conceivable that an intelligent person, least of all a physician or a veterinarian, would hold at the present day that a dog affected with simple indigestion, convulsions, cerebritis, or paralysis would develop virulent saliva capable of causing by inoculation a fatal disease of the nervous system in other animals and with the transmitted peculiarity of virulent saliva. If this fanciful idea were established as a fact the dog would be about the most dangerous animal in existence, and very few of us would care to come in frequent contact with him. So far from making us more tolerant of the dog, the demonstration of the spontaneous development of a deadly virus in this animal's saliva for such slight causes as have been assigned would make us shun and fear him as we do the most venomous serpents. Fortunately for the dog, fortunately for mankind, and fortunately for all animated creatures, this conception of the self-constituted apologists for the canine species is without the least foundation in science, and undoubtedly false.

The spontaneous generation of rabies under certain conditions has been held by many eminent men who would not for a moment entertain the view that the disease developed from indigestion, epileptic fits, and other common ailments. The same men held that rinderpest, pleuro-pneumonia, and glanders originated spontaneously; but how rapidly has the belief in the origin of these diseases in the absence of contagion died out since the discovery of the nature of contagion! Who to-day believes in the spontaneous origin of pleuro-pneumonia or glanders? We know that these diseases are caused by vegetable parasites of the nature of bacteria, and that if the bacillus of glanders or that of pleuro-pneumonia originally had its habitat in nature outside of the animal body, as is probable, the transformation from a saprophytic to a parasitic existence unquestionably required unusual conditions and a long period of time. That the same is true of the contagion of rabies follows from analogy, and is confirmed by the failure of the disease to develop in Australia and by its absence from Sweden and Norway since its eradication from those countries. More recently we have seen the disease in Great Britain reduced from more than six hundred cases a year practically to zero by a few years of intelligent and systematic repression. Surely this result would not have been attained if the disease frequently originates *de novo*. There is no direct evidence in favor of the spontaneous generation of rabies; investigators who have tried to produce it by keeping dogs under the conditions which were said to cause it have uniformly failed. The tendency of modern thought and modern knowledge is unfavorable to this hypothesis, and it is regarded as antiquated, discredited, and unworthy of serious consideration in the absence of direct and convincing evidence. If we admitted the spontaneous generation of the disease, however, such a conclusion would in no sense benefit the dog nor those interested in that animal. On the contrary, it would make us more suspicious of the canine species and require all the more rigorous destruction of superfluous animals, with the strict enforcement of regulations to prevent contagion.

*The Frequency of Rabies.* Passing to the second contention, viz., that rabies is extremely rare, even in animals, and that not one case in a million supposed to be rabies is properly diagnosed, let us briefly inquire into the facts. There are certain European countries which keep very accurate statistics of rabies in animals, and the official reports of which may be taken as fairly reliable. In Germany these reports show 1202 cases of rabies in animals in 1898; in France, 2374 cases in 1899; in Belgium, 444 cases. In Great Britain in 1895 there were 727 cases, and in Hungary in 1895 there were 1397 cases.

In the United States, unfortunately, we do not have general statistics concerning this disease. The writer has recently requested information from the State veterinarians and from veterinary schools. A number of very carefully prepared replies have been received, which are summarized as followed:

Dr. Charles P. Lyman, School of Veterinary Medicine, Harvard University, Boston, 25 or 30 cases in eighteen years. He mentions the case of a policeman in the town of Lynn who was bitten and died.

Dr. W. J. Coates, American Veterinary College, New York, 7 cases a year for twenty-five years.

Dr. H. D. Gill, New York College of Veterinary Surgeons, New York, 8 cases a year for three years.

Dr. Wilfred J. Lellman, New York College of Veterinary Surgeons, 4 cases in his own practice and 2 in Dr. Gill's in one session.

Dr. Robert J. Wilson, Assistant Bacteriologist, Department of Health, New York, has confirmed the diagnosis during the last three years of 40 cases in domestic animals and 3 cases in human subjects.

Dr. Leonard Pearson, Veterinary Department, University of Pennsylvania, estimates 300 to 400 cases in fourteen years. As State Veterinarian he has found a great deal of rabies among farm animals. In many cases diagnosis has been sustained by laboratory tests. In man during last year, 2 cases in Lancaster, 1 in Kennett Square, 1 in Philadelphia, 1 in Allegheny. Three years ago a prominent veterinarian of Pennsylvania died of rabies.

Dr. J. M. Wright, McKillip Veterinary College, Chicago, from January 1, 1900, to April 5, had seen 11 cases in the dog and 3 in the horse. Handles about 20 cases of rabies a year.

Dr. A. H. Baker, Chicago Veterinary College, Chicago, has had in the last year about 10 cases in horses and 50 in dogs.

Dr. James Law, New York State Veterinary College, Ithaca, N. Y., has had brains of rabid animals repeatedly sent there for diagnosis, from which successful inoculations have been made. He brought a piece of the medulla of Neil, the keeper of the dog-pound at Newark, N. J., who died as the result of being bitten by a rabid dog, and inoculated a dog and a number of rabbits, some on the brain, others subcutaneously. All developed rabies. Has the best evidence of a number of men who died of rabies and from whom inoculation with saliva or brain substance caused rabies in the lower animals.

Dr. S. Stewart, Kansas City Veterinary College, says that 11 or 12 cases have been brought to the hospital within three years, 5 within one year.

Dr. John J. Repp, Veterinary Department, Iowa State College, says Dr. J. R. Sanders, of Corydon, Iowa, has noted 18 deaths of cattle with rabiform symptoms, 7 out of one herd of fifty. The medulla of one of the seven was sent to Dr. Repp, who inoculated a rabbit, which died in eighteen days, after suffering four days with gradually increasing paralysis.

Dr. H. J. Detmers, Columbus, Ohio, formerly of the Ohio State University, has seen 3 cases in dogs and 1 in a horse since 1893.

The Health Department of Buffalo reports a recent outbreak, not yet entirely over, in which it investigated, on complaint, 45 cases in dogs, and that in addition 74 cases of dumb rabies and 41 of furious rabies were brought to the pound. Animals other than dogs to the number of 27 were affected, and four persons died of the disease. Rabbits and dogs were inoculated experimentally and died of the disease.

Dr. A. W. Bitting, Veterinarian of the Indiana Agricultural Experiment Station, reports one outbreak some years ago. Two or three dogs and several sheep and hogs belonging to the station were affected. One year ago 1 dog, 7 horses, and 8 cattle died; this year, 1 dog.

Dr. C. A. Cary, Agricultural and Mechanical College, Alabama, states 24 cases have been observed there.

Dr. J. W. Scheibler, State Veterinarian, Kentucky, has seen about 20 cases.

Dr. George H. Bailey, State Veterinarian, Maine, had 1 case of dumb rabies in his practice in 1892. States that the Maine General Hospital had 1 case in a young man several years ago.

Dr. A. W. Clement, State Veterinarian, Maryland, reports about 30 cases brought to his attention officially.

Dr. Samuel S. Buckley, Veterinarian of the Maryland Agricultural Experiment Station, reports that at College Park, Maryland, several years ago a collie dog bit three cows, a calf, and a cat, all of which developed the disease.

Dr. Cooper Curtice, State Veterinarian, North Carolina, reports having noted 1 case in the human subject since he has been in that State.

Dr. W. H. Dalrymple, Louisiana State University and A. and M. College, has seen 1 typical case in a horse and at least 6 in cattle. Was informed by Dr. J. W. Dupree, Surgeon-General of the State, that the latter has had 3 cases in the human subject in his practice. In all cases the dogs which did the biting were preserved and died after showing characteristic symptoms of rabies.

Dr. F. A. Bolser, State Veterinarian of Indiana, reports three outbreaks of rabies in six years, affecting horses, mules, cattle, and hogs. Two young men were bitten, badly lacerated, and died in great agony.

Dr. H. P. Clute, State Veterinarian, Wisconsin, reports 14 cases in dogs, sheep, cattle, and horses. Rabbits inoculated at the State Experiment Station from the brain of a dog died of rabies in eighteen and nineteen days; others inoculated from the brain of a calf died of rabies in twenty-one and twenty-two days.

Dr. A. T. Neale, Director Delaware Agricultural Experiment Station, mentions death from rabies of 3 or 4 cows in one herd. Dogs and rabbits were inoculated from the medulla of one of these cows and developed the disease. A dog at the station which was bitten by a strange dog became affected with rabies. Diagnosis was proved by inoculating rabbits. One case, in a horse, not tested by inoculation.

Dr. M. E. Knowles, State Veterinarian, Montana, has seen about 60 cases of rabies in fifteen years, of which 53 were brought to his attention officially.

Dr. J. W. Elliott, State Veterinary Surgeon, South Dakota, has had as many as 100 cases brought to his attention in the last two years, mostly in cattle, and the disease could be traced to the bites of affected dogs.

Dr. G. T. Seabury, State Veterinarian, Wyoming, has seen 3 cases in dogs. He knows of a case in man caused by the bite of a skunk.

Dr. Sol. Bock, State Veterinary Surgeon, Colorado, has seen at least 50 cases in the last year.

Dr. Paul Fischer, Kansas State Agricultural College, reports 1 case in a horse in 1897. Rabbits inoculated died in thirty days of rabies.

Dr. A. T. Peters, Animal Pathologist, University of Nebraska, reports about eight different outbreaks recorded there. In one outbreak a dog bit several other dogs, a cow, and a horse. The cow was quarantined and died thirty-one days afterward of rabies. The horse was but slightly wounded and developed rabies after about two hundred days.

Dr. L. L. Lewis, Department of Veterinary Science, Oklahoma Agricultural and Mechanical College, states that 2 cases of rabies have come under his observation since he has been in that position.

In the District of Columbia the first investigations by the Bureau of Animal Industry were made in 1893; they were discontinued during 1894, and were taken up again in the fall of 1895, since which time cases of canine rabies have been demonstrated every year. There have been sent to the laboratory by the Health Department a number of dogs each year that were suspected of rabies.

As all such cases which have occurred have not been sent to the laboratory, it cannot be said that we have discovered all of the cases of rabies which have occurred. The number of demonstrated cases of rabies in dogs in the District of Columbia is as follows: In 1893, 11 cases; in 1895, 2 cases; in 1896, 5 cases; in 1897, 3 cases; in 1898, 7 cases; in 1899, 19 cases; in 1900 to July 31st, 29 cases, making a total of 76 cases in dogs. All of these cases have been tested by inoculation, and have given positive results. In addition to these figures a certain number of other animals have died of rabies.

Seven deaths of persons from rabies are recorded by the Health Department of the District of Columbia since 1877. In 2 cases the diagnosis was proved by rabbit inoculations.

These figures do not indicate that rabies is such a rare disease as is alleged. So far from its being true that not one case in a million supposed to be rabies is really that disease, we have found more cases of rabies than were supposed to occur. Not even the most sensational newspapers in our city have had as many reports of rabies in dogs as were actually found by these tests.

